# Multifaceted Sexual Dysfunction in Dialyzing Men and Women: Pathophysiology, Diagnostics, and Therapeutics

**DOI:** 10.3390/life11040311

**Published:** 2021-04-02

**Authors:** Jadzia Chou, Thomas Kiebalo, Piotr Jagiello, Krzysztof Pawlaczyk

**Affiliations:** Department of Nephology, Transplantology and Internal Medicine, Poznan University of Medical Sciences, 60-355 Poznan, Poland; jadzia.chou@gmail.com (J.C.); thomas.kiebalo@gmail.com (T.K.); piotr.jagiello1@gmail.com (P.J.)

**Keywords:** renal replacement therapy, dialysis, CKD, sexual dysfunction, fertility, quality of life, Female Sexual Function Index (FSFI), International Index of Erectile Function (IIEF), erectile dysfunction, renal transplant

## Abstract

Patient survival continues to increase with the growing quality of dialysis and management of chronic kidney disease (CKD). As such, chronic therapy must include considerations of quality of life (QOL), and this includes the disproportionate prevalence of sexual dysfunction (SD) in this patient population. This review aims to describe the pathophysiological and the psychosocial causes of SD with regard to renal replacement therapy, particularly hemo- and peritoneal dialysis. The differences in its manifestation in men and women are compared, including hormonal imbalances—and therefore fertility, libido, and sexual satisfaction—the experience of depression and anxiety, and QOL. The impact of comorbidities and the iatrogenic causes of SD are described. This review also presents validated scales for screening and diagnosis of SD in CKD patients and outlines novel therapies and strategies for the effective management of SD. Increased prevalence of CKD invariably increases the number of patients with SD, and it is crucial for health care professional teams to become familiar with the clinical tools used to manage this sensitive and under-quantified field. As a known predictor of QOL, sexual function should become a point of focus in the pursuit of patient-centered care, particularly as we seek to achieve as “normal” a life as possible for individuals who receive dialysis.

## 1. Introduction

Few studies have been conducted exploring sexual dysfunction (SD) in chronic kidney disease (CKD) despite its high incidence; up to 75% of dialyzing men report erectile dysfunction (ED), while 30–80% of women report sexual symptoms [1]. The impact of SD on quality of life (QOL) is well recognized and is associated with low self-esteem and confidence and higher rates of anxiety and depression [2]. As advances in dialysis therapy decrease mortality in CKD and end-stage renal disease (ESRD), the identification and the management of SD in both men and women remain under significant scrutiny as we seek to achieve as “normal” a life as possible for individuals who receive dialysis.

In this review of recent literature, the current knowledge surrounding pathophysiological and psychosocial factors governing SD in dialyzing men and women is discussed with special consideration for the overall QOL. The impact of common comorbidities and the iatrogenic causes of SD are addressed. Furthermore, we make a review of the management and the novel treatment strategies of SD in men and women and make note of the crucial role of health care professional teams in the chronic care of CKD patients.

### 1.1. Epidemiology

In 2017, 697.5 million cases of CKD were recorded, representing a global prevalence of 9.1% (8.5–9.8) [3]. In 2020, the number of individuals receiving renal replacement therapy (RRT) worldwide was estimated to be 2.5 million persons, with an estimated growth to 5.4 million by 2030 [3]. Described as a “miracle to mainstream” therapy, dialysis is now the mainstay for irreversible renal failure. This transition has been attributed to the increased availability of dialysis, the aging population, the increased prevalence of diabetes mellitus and hypertension, and environmental toxins [4].

RRT is provided in three main modalities: hemodialysis (HD), peritoneal dialysis (PD), and renal transplantation (RT). In HD, the patient’s blood is filtered at an in-center setting three times a week using a dialyzer that removes excess fluid and toxins. The majority of dialysis patients worldwide receive HD (89%), and 90% of these HD patients reside in high-income or upper-middle income countries [4]. One subtype of HD is nocturnal hemodialysis (NHD), in which longer and more frequent HD sessions are organized in home and in-center settings [5]. While it appears to result in improved patient outcomes and better physiological benefits, NHD is fairly underutilized [6]. In contrast, PD utilizes the patient’s own peritoneal membrane to remove toxins and fluids from the body. The patient can administer their therapy at home by themselves or with the help of a care-giver. The main forms of PD are continuous ambulatory PD (CAPD) and automated PD (APD). PD is available in approximately 75% of countries worldwide, and considerable differences exist between territories due to governmental policies and the density of HD facilities [4].

RT is the optimal treatment for ESRD, leading to considerable improvement in QOL and decrease in mortality. As with many organs, the demand outweighs the supply, and waitlist times continue to be long [7]. The most important goals after RT are patient and graft survival, and these are generally achieved through immunosuppressive treatment and the management of infections, malignancies, and cardiovascular comorbidities [8].

Currently, both HD and PD are effective methods of dialysis and show comparable 1 and 5 year survival rates [9]. The high cost of HD has led many low-to-middle-income countries to adopt a PD-first policy [10]. As the prevalence of CKD increases, so will the prevalence of RRT, and health care providers must be aware of the perceived health care needs of this heterogeneous patient population. As survival increases, clinical focus should shift to patient-centered care and seek to improve QOL, of which sexual health is a substantial component.

### 1.2. Hormonal and Gynecologic Considerations in Dialyzing Women

Women with chronic kidney disease clinically present with a high prevalence of anovulation, infertility, menstrual irregularities, and premature menopause. The hormonal and the gynecologic components of these abnormalities have been explored extensively, but several pathophysiological factors remain the subject of ongoing investigation [11].

Here, we summarize the normal physiology of ovulation and menstruation before discussing aberrations in the course of CKD. A typical menstrual cycle is 28 days in length and consists of follicular, ovulatory, and luteal phases. Gonadotropin-releasing hormone (GnRH) is secreted by the hypothalamus and regulates the secretion of follicle-stimulating hormone (FSH) and luteinizing hormone (LH); the former is responsible for the maturation of select follicles between the late luteal phase of the previous cycle and the early follicular phase, whereas the latter increases throughout the follicular phase and responds to the concentration of estradiol. When estradiol peaks on approximately day 8 of the cycle, positive feedback from this increased and sustained level prompts the “LH surge”, which induces ovulation. Progesterone concentration, which starts to climb immediately prior to the LH surge, continues to increase due to its release from the corpus luteum. Estradiol begins to decline following ovulation, and in the absence of fertilization, progesterone also begins to decline at approximately day 21. Disruption of this hormonal pathway results in pathophysiological consequences.

It should be noted that CKD itself impacts the hormonal profile, with dialysis only exacerbating the imbalances. In the discussion of general pathophysiologic mechanisms governing SD, comparisons between the outcomes of the different RRT techniques—renal transplant, nocturnal hemodialysis, continuous ambulatory peritoneal dialysis, and conventional hemodialysis—are made where possible.

#### 1.2.1. Hormonal Imbalances

Anovulation is common in premenopausal women with CKD and/or receiving dialysis and likely results from inhibition of the estradiol-stimulated LH surge [12]. In these women, estradiol fails to rise as it should during the follicular phase, and the administration of exogenous estrogens does not stimulate the preovulatory LH peak. Basal body temperature does not increase during the ovulatory phase, and there is a notable lack of luteal phase endometrial development [11,13,14]. Women with CKD also tend to enter menopause approximately 5 years earlier than non-uremic women [15], and premature ovarian failure is reported to be 3.9%, compared to the 1% prevalence in the general population [16].

Hyperprolactinemia is also characteristic in this cohort and is most likely due to autonomous hormone overproduction [11] and decreased metabolic clearance rate [17]. In CKD, its secretion is resistant to stimulatory (e.g., dopamine infusion) or inhibitory (e.g., insulin-induced hypoglycemia, arginine infusion) actions, and resolution can be appreciated after RT [11]. Hyperprolactinemia has been associated with infertility and decreased libido and in women is also known to contribute to amenorrhea or oligomenorrhea and galactorrhea [18]. The arcuate nucleus neuropeptide kisspeptin has recently been identified as a key driver of pulsatile GnRH release, thus “GnRH pulse generator”, and is considered critical for fertility in humans. Significantly, it is the most potent stimulator of GnRH activity discovered to date [19,20]. Further research has proven that prolactin-induced suppression of GnRH secretion is mediated by the inhibition of kisspeptin neurons [20,21], but interestingly, kisspeptin itself has been shown to stimulate prolactin secretion through the Kiss1r receptor and other pathways [20]. A study by Luedde et al. [22] found that serum kisspeptin in ICU patients was significantly elevated and strongly correlated with markers of kidney injury such as decreased glomerular filtration rate (GFR) and serum creatinine. The authors suspected that this was related to the decreased renal elimination of the neuropeptide. It then stands to reason that an accumulation of serum kisspeptin as a result of impaired renal function and renal failure may have implications for the GnRH reproductive axis derangements previously described. No studies exploring this hypothesis were found in the literature, and the role of kisspeptin in other tissues is still the subject of active research [23].

In concert, these hormonal imbalances of the hypothalamic–pituitary–gonadal (HPG) axis are thought to be the primary causes of menstrual irregularity, low sex drive, and infertility in female CKD and dialysis patients. The intricacies of the numerous feedback pathways are areas of ongoing study and are beyond the scope of this review.

#### 1.2.2. Anemia and EPO Therapy

The prevalence of anemia in the dialyzing patient is as high as 90% in some studies [24], and data show that its occurrence increases stepwise with decline in GFR [25]. Typically normocytic, normochromic, and hypoproliferative, the development of anemia in CKD is complex [24]. Relative decrease in erythropoietin (EPO) production by renal peritubular fibroblasts leads to defective erythropoiesis, resulting in abnormal maturation of red blood cell (RBC) precursors. Erythropoiesis may also be stunted by uremic-induced inhibitors such as indoxyl sulphate (IS), indole-3-acetic acid (IAA), and paracresyl sulphate (PCS). IS has been implicated in the impairment of erythropoiesis through various mechanisms, such as limiting EPO gene transcription [26], stimulating hepcidin production, leading to iron sequestration [27] and possibly triggering suicidal RBC death [28]. CKD patients also suffer from functional iron deficiency, the so-called “anemia of chronic disease” characterized by normal or high serum ferritin (i.e., adequate iron stores) and low serum transferrin saturation (i.e., low circulating iron) [24]. Furthermore, anemia in spite of high EPO concentration suggests some mechanism of peripheral hyporesponsiveness in the course of CKD [25].

Anemia is exacerbated by dialysis therapy, with increased loss of iron resulting from platelet dysfunction, hemolysis, and blood retention in the extracorporeal circulation. While uremic toxins worsen anemia parameters in CKD, there was no correlation between dialysis and IS, IAA, or PCS (Bataille et al., 2017). There is scant information on anemia and the treatment of anemia in PD patients [29]. The prevalence of anemia remains high after RT at approximately 36–40% [30,31] and is associated with increased mortality [31,32].

In addition to the iron losses of dialysis, dialyzing patients, in particular HD patients with high CRP, also suffer from impaired absorption of dietary iron [33]. It is well established that iron-deficiency anemia (IDA) leads to fatigue, anxiety, weakness, and impaired physical and mental capacity [34]. For these reasons, IDA is thought to be a significant factor reducing sexual function in women [35]. Gulmez et al. [36] showed that iron supplementation in 207 women with IDA significantly enhanced sexual performance score as well as improved physical and social function while decreasing anxiety and depression. In contrast, Hartmann et al. [37] report in a review of 77 eligible articles that they were unable to find any correlation between IDA and female SD. Inconsistencies in the literature may be due to a variety of reasons including age of the surveyed cohorts and terminology and survey methods. The mean age of Gulmez et al.’s cohort was 33.6 ± 8.4 years, while Hartmann et al.’s inclusion criteria were women between the ages of 45 and 65; Gulmez et al. used the Female Sexual Function Index (FSFI) to quantify SD, while Hartmann et al. searched multiple databases using a variety of pertinent keywords [36,37].

The treatment of CKD-associated anemia is with erythropoiesis-stimulating agents (ESAs)—recombinant human erythropoietin (rHuEPO) such as epoetin alpha and epoetin beta—and iron supplementation [38]. Approved by the FDA in 1989, rHuEPO therapy was immediately shown to increase cognitive function, exercise tolerance, sexual potency, and QOL in HD patients [39]. Since then, rHuEPO has been associated with improvement in sexual function and libido in women and men [40,41]. EPO therapy also appears to correct imbalances in sex hormone profile in men [42,43] and women [40]. However, it has since been shown that long-term use of short-acting ESAs increases the risk of adverse cardiovascular outcomes and vascular access thrombosis [44]. Long-acting ESAs increase mortality in HD patients [45]. Li and Wang [46] showed in a large observational study that PD patients required lower doses of ESAs to reach target hemoglobin levels, which may be significant considering the emerging safety concerns.

Like with many medications, a proportion of patients are low- or non-responders, and the same has been observed with EPO therapy. This is quantified by the EPO resistance index (ERI). Among HD patients, approximately 5–10% display hyporesponsiveness to EPO, and this is related to inflammation status, nutritional status, and dialysis adequacy [47]. There are fewer investigations of EPO hyporesponsiveness in chronic PD. In their study of 14 PD patients, Hara et al. identified fluid overload as the key independent predictor, contrary to previous studies on the subject that identified CRP and hyperparathyroidism. Makruasi et al. [48] conducted a comparable study on a larger patient group; they found that EPO hyporesponsiveness was 6.7% in their PD patient cohort, and that female gender was the predictive factor. In a large comparative study of PD patients, Ryta et al. [49] found that women had significantly lower hemoglobin levels despite higher dialysis adequacy and higher doses of ESAs, and that gender was an independent determinant of ERI. Further large-scale cohort and comparative studies are required to explore the potential reasons for these findings.

#### 1.2.3. Menstrual Disturbances

There is also no overall consensus in the literature as to the typical menstrual pattern of a CKD patient and/or on dialysis, and the overarching assessment is that the cycle is almost invariably irregular once the GFR falls below 15 mL/min [50]. Among the limited number of studies, there is considerable variability. Guglielmi et al. [18] also points out that the majority of relevant papers were published before EPO became an established treatment in the course of CKD.

To reference only a few studies, menstrual disturbances are reported to occur in the range of 64.2% (of 165) [51], 73% (of 63) [52], 74% (of 39) [53], and 80% (of 25) [54] of premenopausal patients after the initiation of dialysis. Of these, Lin et al. [51] has documented the largest cohort of 165. The most common disturbance is secondary amenorrhea [51,52,53,54]. In a small cross-sectional analysis of 30 chronically dialyzed women, only half eventually achieved stable frequency, regularity, duration, blood loss, and pain [55]. The cross-sectional study by Lin et al. also compared the overall prevalence of menstrual disturbances between four RRT modalities, and the results were as follows: RT (50%), NHD (55%), CAPD (72.1%), and HD (76.1%) [51]. Regular menstruation often reestablishes after RT [16,51,53]. Menses are also well documented to reestablish after the initiation of PD [56].

In the study by Lin et al. [51], hormonal profiles were also evaluated between the four RRT modalities. Serum prolactin was significantly lower, and serum progesterone was significantly higher in the RT and the NHD groups. Furthermore, LH, FSH, and testosterone profiles were also found to be closer to normal in RT and NHD groups compared to HD and CAPD groups [51]. That NHD may restore normal hormonal milieu more effectively than HD is supported by previous, albeit very small, cohort studies [12,57], and numerous other studies have found that RT results in appreciable normalization of sex hormone profiles [58] and improvement of self-reported sexual function [16,53,58,59].

#### 1.2.4. Fertility

Anovulation and abnormal menses are associated with CKD, ESRD, and dialysis as described above. Primary ovarian insufficiency (POI)—the cessation of menses at <40 years of age—is reported by 3.9 to 20% of premenopause-aged women with ESRD, but whether this is truly premature menopause is hard to define [60]. Menopause is a permanent state achieved when the number of remaining follicles approaches 1000; however, the rate of ovarian follicle atresia in CKD or post-RT is not known. Secondly, since RT, hormone replacement therapy, bromocriptine, and/or frequent dialysis may restore menses, long-standing cessation of menses may be a relative state [60,61]. Regardless, fertility in women with ESRD is estimated to be reduced 10-fold [62].

The number of successful and healthy pregnancies in dialyzing or post-transplant women has increased [63]; however, the likelihood of conception and successful pregnancy remains low. In women undergoing HD, the increase in reported pregnancies has been significant. The Australian and New Zealand Dialysis and Transplant Registry recorded 0.67 pregnancies per 1000 person-years (1986–1995), which has now increased to 3.3 pregnancies (1996–2008). This has been attributed to intensified HD regimens (including nocturnal therapy), increased use of EPO, and increased clearance. In contrast, the same registry reports that women receiving PD have a conception rate of only 1.06 pregnancies per 1000 person-years, and the reason remains unclear [64]. Hou [65] and Dimitriadis and Bargman [56] both propose that peritoneal dialysate obstructs the passage of the ovum to the Fallopian tubes. It is not established if women who wish to conceive should be switched from PD to HD, though it is the practice in certain centers [64].

RT restores fertility; however, pregnancy after RT is considered high risk, and transplant recipients are at higher risk of maternal complications such as tissue rejection, graft loss after pregnancy, preeclampsia, hypertension, and urinary tract (and other) infections. Expectant mothers are managed by a multidisciplinary team of high-risk obstetricians, perinatologists, and transplant nephrologists, and women of child-bearing age should be fully informed and counseled by the transplant team [62].

#### 1.2.5. SD and Comorbidities

There is a strong association between SD and diabetes mellitus (DM), but the studies investigating the effect of DM on female sexuality have yielded inconsistent results [66]. One study suggests that DM results in a decrease in genital sensation in women [67]. Hypertension and/or antihypertensive medication have also been associated with SD. A recent meta-analysis revealed considerable heterogeneity in the literature, with the prevalence of SD in hypertensive women varying between 14.1 to 90.1% [68]. Furthermore, it was acknowledged in a systematic review [69] and the Third National Survey of Sexual Attitudes and Lifestyles [70] that hypertensive women exhibited higher prevalence of SD than normotensive women. A number of papers have identified that first and third-generation beta-blockers as well as a whole host of antihypertensive drug classes are associated with decline in sexual function; however, it is not clear if SD precedes the antihypertensive treatment or occurs after [69,71,72]. Iatrogenic drugs are discussed in more detail below.

### 1.3. SD in Dialyzing Women

It has been accepted for some time that the somatic mechanisms described above do not adequately explain the high rate of SD in male and female patients with CKD and/or RRT [1,73,74]. It is now well established that SD is a major determinant of life quality and is associated with anxiety, depression, social withdrawal, and loss of self-confidence and self-esteem [2]. However, compared to men, the clinical research and investigation of SD in women on different RRT modalities is lacking [75,76]. Furthermore, the lack of clear definition of female SD has resulted in inconsistencies in the literature [2], and we begin with a brief comment below.

#### 1.3.1. “SD” vs. “Sexual Problems”—The Limitations of the FSFI Instrument

It should be noted that there are some general criticisms concerning studies on female SD. Firstly, previous studies may utilize disease definitions that have since been rendered obsolete. For example, Toorians et al.’s publication [74] is widely cited, but the authors investigate several sexual disorders that have been removed from the DSM-V, such as sexual aversion disorder. Secondly, there is no clear differentiation between somatic dysfunction and subjective sexual function. Mor et al. have termed the former “true SD”—that is, physiologic difficulties during sexual intercourse and separate from sexual inactivity, satisfaction, and so on [77]. Diemont et al. [78] also make a similar distinction: “SD” being a disturbance in the sexual response cycle and “sexual problems” being the subjective evaluation of sexual function. The gold standard measure of female sexual function, the Female Sexual Function Index (FSFI), itself does not distinguish between the two components of sexual health and experience. The FSFI contains 19 questions pertaining to the quality of sexual intercourse, desire, intercourse satisfaction, lubrication, ability to achieve orgasm, and degree of clitoral sensation [79]. All sexual inactivity is scored to equate to SD [80], and there have been concerns raised over the inclusion of zero in some but not all response scales [81]. As a significant limitation of an otherwise reliable and valid assessment tool, clearer distinctions need to be made for the inclusion and the exclusion of sexually active and inactive participants [82]. In a study by Mor et al., [77], a modified FSFI was administered monthly to 125 HD patients over 6 months. In total, 81% of the cohort was sexually inactive for reasons such as lack of interest (43%) and lack of a partner (39%); sexual difficulty as a primary cause was only reported by 2% of respondents. Probative questions further revealed that, of the women reporting lack of interest in sexual activity, 76% were moderately to very satisfied with their sexual life, indicating that, while sexual inactivity may be interpreted as SD, it was not the case in this cohort. These findings are consistent with other large cohort studies [83,84], indicating that sexual dissatisfaction and sexual difficulty in women on chronic HD may be much less prevalent than previously suggested.

Notably, Diemont et al. sampled a large Dutch cohort without use of the FSFI. While erectile dysfunction is highly prevalent in male dialyzing patients, the authors note that, in women, subjective sexual arousal is related more to the setting of the sexual stimulus rather than genital arousal, which means that somatic problems in women—i.e., “true SD”—may have less influence on the occurrence of a sexual problem [78]. This may support the apparently low prevalence of sexual difficulty compared to subjective SD reported by Mor et al.

Regardless of these discrepancies, notable and significant differences still exist when women receiving RRT are compared with healthy population control [75]. Yazici et al. identified with the FSFI that 94.1% of their PD cohort and 100% of their HD cohort experienced SD compared to 45.8% of control. Several research teams showed that female patients receiving PD reported better sexual function compared with those receiving HD [66,74,75,78,85]. A small study by Duarsa et al. [86] also showed that women undergoing CAPD experienced a significant improvement in FSFI scores. This may be due to the fact that PD patients have lower incidence of anemia, better clearance of middle molecular-weight substances, and better residual renal function. The increased independence of a PD patient and fewer dietary restrictions may have a positive impact on psychological wellbeing [66]. In contrast, amenorrheic HD patients are more likely to have low estradiol levels secondarily leading to vaginal atrophy and dryness [11] and therefore exhibit higher prevalence of dyspareunia and problems with orgasm [2].

Toorians et al., as with Mor et al., note that SD in women, regardless of the type of RRT, seems largely due to loss of sexual interest—fatigue, listlessness—rather than erectile failure (in men) or metabolic disturbances (in women). They conclude that, most likely, it takes more than erectile potency or vaginal engorgement for the individual to perform sexually [74]. As such, it is perhaps more difficult to study female SD compared to male, owing in part to culture and the subjective self-reporting methods that do not fit the medical model as well as objective measures such as penile erection quality and ejaculation latency time [87]. These factors significantly contribute to the difficulties in quantifying this field of study.

#### 1.3.2. Depression, Anxiety, and Quality of Life

Psychological factors have been heavily investigated for this very reason. In the study by Mor et al., the women who were—according to FSFI—sexually dysfunctional reported similar QOL and depression scores to women without FSFI-defined SD [77], but the association in the literature between SD and depression and QOL varies [73,84,88]. Depression can be evaluated using a variety of evidence-based measures including Beck’s Depression Inventory (BDI), the Center for Epidemiologic Studies Depression Scale (CES-D), the Patient Health Questionnaire-2 (PHQ-2), and the hospital anxiety and depression scale (HADS), all of which are validated for and commonly used in patients with ESRD [75,88]. Considerations are made for different cultures, and cut-off scores reflect the psychiatric heterogeneity between populations. QOL is similarly assessed using the World Health Organization Quality of Life (WHOQOL-BREF) instrument, and Medical Outcomes Study short form (SF-36) also validated for patients with ESRD [75,89].

In accordance with findings from the PRESIDE study, depression and anxiety are significantly correlated with SD and sexual inactivity [87]. Yazici et al. [75] reported that there was a significant negative correlation between total FSFI score, BDI, and the mental-physical component score of QOL for both PD and HD female patients. In total, 75.3%, 43.8%, and 4.2% of PD, HD, and control subjects, respectively, were depressed in their study. In contrast, Koca et al. [66] found that, while significantly more women receiving HD, PD, or RT were depressed compared to control, there was no significant difference in depression scores between the three RRT techniques. Saglimbene et al. [90] analyzed 232 sexually active women on HD. They found that depression was correlated with worse lubrication and pain scores as assessed by the FSFI instrument, and that pain and low sexual function appeared to be associated with comorbidities such as previous cardiovascular events. No similar study was found for female PD patients, who generally suffer lower rates of vaginal atrophy and dryness than HD patients.

Recent studies have improved upon the limitations of observational data by using a mixed-methods approach to assess patient-reported outcome measures. A systematic review by Budhram et al. [91] assessed how HD and PD impacted specific QOL domains in nine eligible studies. They found that domains including “sexual function” and “role limitation due to physical function” were scored better in PD, while domains such as “body image” and “overall health” favored in-center HD. Tannor et al. [10] found no overall difference in kidney disease quality of life (KDQOL-SF) scores between HD and CAPD male and female patients, but qualitative analysis revealed that their CAPD patients reported higher symptom burden, reduced social and sexual functioning, and poor energy/fatigue compared to HD, contrary to the findings by Budhram et al. [91] and other similar studies. Tannor et al. note that their Cape Town center is not well resourced, and that their findings may be reflective of RRT programs in resource-limited settings.

Anxiety is often experienced together with depression and is often assessed using the State-Trait Anxiety Inventory (STAI 1/STAI 2). State anxiety is the fluid, fluctuating, and temporary emotional state that responds via autonomic stimulation to a specific event, while trait anxiety is a stable attribute of personality and may be associated with psychopathological conditions [92]. The STAI 1/STAI 2 consists of 40 self-reported items, and a high score indicates an anxious individual. Both state and trait anxiety have been negatively associated with subjective and physiological sexual arousal [93], and women with premorbid history of anxiety disorder are four times more likely to suffer from vulvodynia [94]. The potential mechanism is speculated. It is possible that the individual is specifically anxious about her sexual function and performance, and this causes her to respond inadequately to stimuli. It may also be because non-sexual cognitive distractions—obsessions and sensation hypervigilance—interfere. Lastly, because the autonomic pathway governs both arousal and acute anxiety, anxiety may physiologically impair arousal [93].

Theofilou [95] found that sexual function—the subjective assessment of sexual satisfaction—in 144 men and women on HD and PD was negatively associated with STAI 1/STAI 2 score and was also correlated with depression, confirming the strong associations of sexual function with mental health in CKD patients. Their results suggest that HD and PD patients with satisfactory sexual life feel less anxious and depressed and are more likely to evaluate their general health more favorably. Koca et al. [66] also found that RRT groups were more anxious than control, and their PD and RT groups scored higher anxiety than HD and control. A small study by Stasiak et al. [96] found no difference in anxiety between PD and HD patients, whereas Ozcan et al. [97] found that RT recipients were more anxious and depressed compared to HD and PD in the context of CKD.

Depression and anxiety in RRT have also been explored through a variety of parameters. Vitamin D deficiency was investigated as a potential cause of depression in 484 dialyzing men and women. While a greater degree of depression was associated with lower serum vitamin D in these patients, one year calcitriol supplementation did not enhance depressive symptoms [98]. Selvi et al. [99] explored dialysis adequacy as a determinant of sexual function and depression in men and women receiving HD and PD. They found that, in women, dialysis adequacy (Kt/V cut-off values of 1.3 for HD and 1.7 for PD) and depression were significant factors affecting SD. However, reduction in QOL was not observed in their female cohort. A small randomized controlled trial by Dziubek et al. [100] found that physical training and exercise in men and women receiving HD significantly reduced depression and anxiety, and a review by Bohm et al. [101] showed that longer exercise duration (>6 months) in dialyzing adults was correlated with a significant improvement in symptoms of depression.

These recent studies provide a clearer picture of the rate of depression and anxiety in different RRT modalities, highlighting the importance of identification and management of psychiatric comorbidities in the investigation of SD. Some therapeutic strategies offer non-pharmaceutical solutions to a multifactorial problem, allowing for patient-centered tailoring of treatment. The pharmacological management of depression is discussed in greater detail below.

### 1.4. Hormonal Imbalances and SD in Dialyzing Men

While reproductive axis disturbances are more prominent in women, male CKD patients are shown to suffer multi-system pathophysiology that impacts several aspects of sexual health. The most common disturbance in sexual health in the dialyzing man is erectile dysfunction (ED). Other disturbances include testicular damage, decline in testosterone, HPG axis dysfunction, and hyperprolactinemia, culminating in low libido and infertility [11].

As with women, decline in renal function and GFR leads to progressively greater disturbances in the HPG axis [102]. While these disturbances do not generally normalize with HD or PD, RT appears to restore normal sexual activity to some degree [11].

#### 1.4.1. Testicular Damage and Hypogonadism

The pathophysiology of testicular damage in uremic patients is not well understood. Semen analysis typically shows decreased ejaculate volume and low motility, and histopathology reveals reduced spermatocytes and poor sperm maturation. These findings are present before the initiation of dialysis, and the condition generally deteriorates with the progression of therapy [11]. Total and free testosterone is reduced in CKD patients, and there is a documented negative correlation between testosterone concentration and CKD stage [102]. Administration of human chorionic gonadotropin (hCG) in an effort to stimulate testosterone yields only a blunted response. In several survey studies, testosterone level only showed a modest correlation with erectile function, if any at all [102], and testosterone replacement in hypogonadal men does not appear to improve libido or potency [103]. It appears that the clinical significance of testosterone level may lie in its role in HPG axis feedback loops. In a cross-sectional study of 79 male patients, Cigarrán et al. showed that patients receiving PD maintained higher testosterone levels than patients receiving HD; testosterone deficiency (<3 ng/mL) was observed in 39.5% of patients receiving HD compared with 5.6% of patients receiving PD. To date, this is one of the few studies to correlate RRT technique with the development of hypogonadism [104].

Testosterone inhibits LH secretion by Leydig cells; excess LH in both men and women is characteristic of CKD and is thought to be due to the lack of feedback inhibition. The accumulation of LH is also likely due to reduced kidney clearance, and this derangement does not appear to correct with dialysis. Together, the described testosterone and LH profile produces hypergonadotropic hypogonadism in advanced CKD [102]. Chronically elevated kisspeptin is known to inhibit spermatogenesis, resulting in testicular degeneration. Several studies demonstrate the potential effects of kisspeptin on testicular health, and chronic administration of the neuropeptide has consistently been shown to produce negative effects [23].

Hyperprolactinemia in uremia is previously described in this review, and its pathophysiology in men is as in women. Its clinical significance remains the subject of study. Extreme hyperprolactinemia in men is associated with low libido, decreased testosterone, and infertility. Hyperprolactinemia may be involved in the pathogenesis of gynecomastia, which affects approximately 30% of men on maintenance HD [11]. As in female dialyzing patients, a small observational study reported that switching from HD to NHD resulted in significant reduction in prolactin and increase in total and free testosterone, while LH and FSH remained unchanged [57]. Further studies are required to confirm this benefit of NHD. Finally, RT appreciably normalizes the male hormonal profile (testosterone, LH, and prolactin), resulting in improvement in sperm motility, density, viability, and morphology [12].

#### 1.4.2. Fertility

Compared to women, male reproductive function and fertility in the course of CKD and ESRD is not well studied. In the general population, key hormonal biomarkers for the assessment of hypogonadism include testosterone, LH, FSH, GnRH (discussed above), as well as anti-Müllerian hormone (AMH), inhibin B, and insulin-like factor 3 (INSL-3) [105].

Anti-Müllerian hormone (AMH) is a high-molecular weight glycoprotein that is secreted exclusively by Sertoli cells at high levels throughout childhood. Its production declines in puberty and adulthood, and its function in adult men is not well known [106]. Low plasma levels of AMH have been associated with decreased fertility and non-obstructive azoospermia. Eckersten et al. [107] reported that dialyzing men have 60% lower levels of AMH compared to healthy fertile control. Another investigation by Eckersten et al. [108] also demonstrated that AMH levels in patients with CKD stages 1–4 showed a similar significant decrease. Due to its high molecular weight, AMH is not removed via glomerular filtration or dialysis, thus its decrease in plasma is suspected to be a result of pro-inflammatory milieu, uremic toxicity, or other causes [108]. As such, decrease in AMH may reflect decline in Sertoli cell function. Eckersten et al. [106] also investigated microRNA (miR)-155—an miR associated with inflammation—as another potential biomarker of male subfertility in the course of CKD. MiR-155 was elevated in men with CKD compared to healthy fertile control, and high miR-15 levels were negatively associated with sperm concentration and total sperm count. MiR-155 levels were independent of CRP and testosterone levels as well as AMH levels. As a result of these studies, Eckersten et al. posit that low AMH and high miR-155 are independent markers of subfertility in men with CKD, reflecting the multifactorial etiology of this dysfunction [106].

Eckersten et al. [107] also investigated inhibin B, a selective FSH inhibitor, in patients with ESRD. In their cohort, there were no differences in inhibin B concentration compared to control. No other studies of inhibin B pertaining to CKD or ESRD were found in the literature. INSL-3, likewise, does not appear to be significant in the course of CKD or ESRD.

Restoration of fertility after RT is a subject of ongoing study. In addition to normalization of hormonal profiles, spermatogenesis and testicular function appears to recover in a small proportion of patients. There is also demonstrated improvement in semen quality, though it is interesting that multiple studies found a decrease in sperm motility post-transplant, with parameters similar to infertile control. It is also postulated that post-transplant immunosuppressive therapy plays a significant role in barring recovery of male fertility, with calcineurin inhibitors and mammalian target of rapamycin (mTOR) inhibitors potentially implicated. There are few studies investigating the impact of these immunosuppressive regimens on male fertility, but RT recipients should be counseled appropriately nevertheless [109].

#### 1.4.3. Impact of Comorbidities

As previously discussed, the prevalence of anemia is high in dialyzing patients, and its development in the course of CKD in men is as it is with women. As with women, EPO deficiency is thought to be the primary cause. In men, testosterone deficiency has been shown to contribute to the increased incidence of anemia [110], and an appreciable improvement in anemia has been observed following androgen therapy [111,112]. This suggests that hypogonadism may contribute to its pathomechanism. Anemia has been correlated with decrease in libido, sexual desire, and erectile function [32,113] with EPO therapy demonstrated to improve sexual desire and erection quality in HD patients [40,41]. As described earlier, anemia remains common after RT.

Furthermore, it is common for patients with CKD to suffer from other chronic diseases such as DM and hypertension, which are independent risk factors for ED [11]. For instance, DM is a leading cause of ESRD and is known to cause vascular, neurologic, and hormonal alterations, which all influence the development of SD [11]. Animal and human studies have demonstrated the decreased production of endothelial-derived nitric oxide in diabetics and the beneficial effect of phosphodiesterase-5 inhibitors, which allows for improved cavernous tissue blood flow [114,115,116]. Furthermore, diabetic neuropathy in men affects key autonomic and somatic nerve processes in the physiology of penile erection [114]. Finally, there has been an observed trend for hypogonadism with concomitant low testosterone in diabetics, which also contributes to SD [117].

#### 1.4.4. Erectile Dysfunction

The most common sexual disturbance in the dialyzing man is erectile dysfunction, with a prevalence ranging between 51.9% [76], 77% [118], 80.6% [119], 83% [120], and 88% [78]. ED is, in itself, multifactorial, resulting from vascular, neurological, and/or psychological derangements in addition to the CKD-related hormone abnormalities [121].

The majority of investigations of ED are conducted on men receiving HD. Generally, the prevalence of ED is reported to be similar between HD and PD patients. A recent meta-analysis reported that, of 7253 patients experiencing ED, 79% received HD, 71% received PD, and 59% were post-RT [122]. Seck et al. [123] reported similar rates of ED in their study group of 70, as did Tekkarismaz et al. [124] in their group of 51 HD and PD patients. Lai et al. [76] reported ED in only 51.9% of their chronic PD cohort, but the authors suspect that this lower prevalence was due to the younger average age in their study group. Recent studies and meta-analyses agree that ED and sexual function appear to improve after RT due to the correction of hormonal imbalances [122,125,126,127]. ED that persists post-transplant is most likely the result of multiple pre-existing comorbidities [125,128], and erectile function after RT remains inferior to that of sexually active, healthy, non-RT population control [129,130]. Systemic organic damage in the course of CKD combined with existing comorbidities and dialysis therapy may be irreversible and unresponsive to RT [128]. Guvel et al. [131] identified calcific changes in the tunica albuginea and epididymis of 20 men (65%) on maintenance HD. As the duration of maintenance HD increases, ultrastructural changes are also seen to develop in the smooth muscle of the corpora cavernosa as a direct result of the uremic state [132]; these vascular calcifications may be only one of many CKD sequelae responsible for persistent ED.

Studies have also compared overall SD between different RRT modalities. Tekkarismaz et al. [124] administered the HADS and the International Index of Erectile Function brief screening version (IIEF-5) to 51 dialyzing men and showed that the frequency of SD was 12.9% with HD and 30% with PD. Jung et al. [89] conducted a mixed-methods cohort study of 989 male HD and PD patients using the kidney disease quality of life (KDQOL) SF^TM^ 1.3 questionnaire, the Short Form health survey SF-36, and the BDI to assess QOL over 24 months over two RRT modalities. PD patients reported higher Health-Related Quality of Life (HRQOL) than HD patients at the initiation of therapy. After 24 months, HD and PD patients experienced worsening of HRQOL in different domains: HD patients in the domains of sexual function and patient satisfaction and PD in the domains of burden of kidney disease, general health, emotional wellbeing, and energy/fatigue. While HD patients experienced worse sexual function, the three questions of the KDQOL related to sexual function are very brief and do not assess sexual (dys)function with the rigor of specialized questionnaires such as the IIEF-5.

#### 1.4.5. Depression, Anxiety, and Quality of Life

SD including ED has a demonstrable impact on QOL, self-esteem, and self-confidence [32,133]. Several evidence-based scales and psychometric measures have been validated for use, and these include the International Index of Erectile Function (IIEF) and its brief screening version IIEF-5 [134], the Sexual Complaints Screener for Men (SCS-M) [135], and the Aging Male Symptom (AMS) scale, which includes questions in a sexual domain [136]. In the evaluation of ED in kidney disease, these are often administered together with depression and QOL questionnaires described above. A recent large multinational cross-sectional study administered the CES-D questionnaire to 946 men receiving HD and found that a higher CES-D score was the strongest correlate of any ED, and that depressive symptoms were strong independent correlates of severe ED [120]. Furthermore, major depressive disorder and various anxiety disorders have been highly correlated with the loss of sexual desire [137]; in particular, social phobia is a recognized risk factor of premature ejaculation, and this pathomechanism may also be rooted in adrenergic hyperactivity, described previously in this review [138].

Depression in this population is multifactorial and is known to be associated with perceived decrease in physical function, loss of occupation or primary family role, and cognitive decline. Additionally, sexual function is recognized to decrease in a bi-directional manner; depression and depressive symptoms may cause ED and interfere with arousal, while unsatisfactory sexual life in turn triggers and/or exacerbates depression [139]. Thus, the amelioration of depression may improve SD and vice versa, highlighting the complexity of sexual and mental health in men with CKD and/or on dialysis [140]. In the large cohort study by Jung et al. [89], PD patients reported better health-related QOL scores than HD patients, including within the domain of sexual function. Oyekçin et al. [141], in their small study group, found that depression and anxiety were more prevalent in HD than PD and control, and that chronic dialysis disturbed body image and decreased sexual satisfaction. Other recent studies also found that HD patients were more depressed and anxious than patients after RT [142,143,144]. In contrast, Peng et al. found no difference in HRQOL between HD and PD patients in a large comparative study in Taiwan [145], and in a similar study of a Taiwanese cohort by Mau et al., only the subscales evaluating bodily pain and social functioning significantly differed between HD and PD patients [146].

Depression and depressive symptoms were identified as significant predictors of mortality in both men and women receiving long-term dialysis [147]. In an investigation by Peng et al. [148], men receiving HD reported lower BDI scores and higher QOL than female HD patients but demonstrated significantly worse survival over 7 years. Depression and anxiety remain underdiagnosed and undertreated in CKD, but more than that, gender differences and their effect on psychological comorbidities and QOL need to be further studied so that clinical care and outcomes may continue to improve for dialysis patients. As an aside, depression is widely treated with antidepressants such as selective serotonin reuptake inhibitors (SSRIs), which, ironically, have a negative effect on libido and erectile function. Potentially beneficial pharmaceutical agents are discussed in detail below.

#### 1.4.6. Other Useful Parameters

Some objective physiological measures may be useful for the clinical determination of SD risk. Residual renal function may be an important parameter when considering QOL in dialysis patients, and it is independently associated with the prevalence of ED, although the mechanism is not clear [119]. Stolic et al. [149] described that dialyzed patients with preserved RRF maintained erectile function better. Ye et al. suspect that, because RRF is preserved better in PD than in HD, this may have implications for ED in PD patients. Furthermore, unlike in PD, RRF is not routinely monitored in HD patients despite standardized guidelines and the evidence that preserved RRF is associated with better outcomes. The use of RRF as a parameter in diagnosis, treatment, and management of ED and SD is still an open question [150].

Dialysis adequacy is another identified parameter. Selvi et al. [99] investigated the relationship between sexual function and dialysis adequacy in men and women receiving HD and PD. This determinant (Kt/V cut-off of 1.3 for HD and 1.7 for PD) was more decisive in men than in women and was strongly related to physical, social, and general health functioning. The authors also found that non-adequate dialysis was negatively correlated with QOL in men but not in women.

In a comparative study by Hassan et al. [121], high peritoneal glucose load index (PGLI) in male patients receiving maintenance PD was correlated with higher prevalence of ED and depression. Patients with PGLI > 3 g/day reported significantly poorer sexual function and lower serum total testosterone. Hassan et al. [151] also investigated the correlation between overhydration in 39 men receiving HD and SD and found that overhydrated patients (OH/ECW ratio > 0.15) had lower IIEF scores and higher rates of depression as well as lower serum total testosterone and dehydroepiandrosterone (DHEA), both of which contribute to sexual interest, arousal, and activity. More large-scale studies are needed before further conclusions can be drawn.

### 1.5. Screening and Diagnostics

The diagnosis of SD in men and women begins with a comprehensive sexual history and physical examination. Adjunctively, an evidence-based questionnaire may be useful to identify SD. There are a variety of instruments that have been validated for use in patients with CKD.

For women, one of the most widely administered is the Female Sexual Function Index [73,75,85]. The FSFI is a self-reported assessment of six domains: sexual desire, sexual arousal, lubrication, orgasm, satisfaction, and pain/discomfort [79]. The limitations of the FSFI instrument are discussed in detail in this review, but it is otherwise considered the gold standard and has been validated in multiple languages [81]. In men, the International Index of Erectile Function and its screening version IIEF-5, also called the Sexual Health Inventory for Men (SHIM), are widely used in clinical and research settings [79,152]. The IIEF is scored in the following domains: erectile function, orgasmic function, sexual desire, intercourse satisfaction, and overall satisfaction [134]; the Erectile Function (EF) domain is of special value as its use as a sole measure can accurately discriminate between men with and without ED [153]. IIEF also demonstrates high sensitivity and specificity and is validated in multiple languages [153].

The Arizona Sexual Experience Scale (ASEX) has also been validated for use in CKD and ESRD patients [154]. This instrument quantifies libido, arousal, vaginal lubrication/penile erection, ability to reach orgasm, and satisfaction from orgasm in patients with psychiatric and/or physical health complaints [155]. The sensitivity and the specificity of the ASEX are 82 and 90%, respectively [156], and may also be valuable as a screening tool [157].

These three instruments are described in further detail in Table 1.

Other validated instruments that have not be verified in CKD and/or dialyzing patients include the Changes in Sexual Functioning Questionnaire (CSFQ) [158], Sexual Interest and Desire Inventory (SIDI), the Elements of Desire Questionnaire (EDQ), the Decreased Sexual Desire Screener (DSDS), and the Female Sexual Distress Scale-Revised (FSDS-R), to name only a few [81].

### 1.6. Therapeutics and Treatment

SD in male and female dialysis patients is multimodal and, as such, treatments should be directed to the most likely cause(s). With a greater appreciation that SD develops not only because of physiologic disturbances but also due to psychological factors, special emphasis should be placed on the recognition and the management of a wide variety of etiologic entities [159].

#### 1.6.1. Iatrogenic SD

The treatment of comorbid conditions may result in SD (i.e., iatrogenic SD); thus, a thorough medication review should be conducted before any proposed therapy may commence [159]. One such comorbid condition is hypertension (HTN) [160]. In women, the overarching question is whether SD precedes or results from antihypertensive therapy. A study of 211 hypertensive women found that SD was likely to be iatrogenic to antihypertensive medication [71]. The 2016 SPRINT trial showed that, while SD had a high prevalence among the sexually active participants, no particular class of antihypertensives had special impact on sexual function [161], and these findings were mirrored in a 2019 systematic review, where the authors found no difference between treatment with alpha-blockers, first and third-generation beta-blockers, calcium-channel blockers (CCBs), angiotensin-converting-enzyme (ACE) inhibitors, angiotensin receptor blockers (ARBs), or diuretics in increasing the risk of SD in women [69]. In a seven patient case series, spironolactone was associated with the development of hormonally-associated vestibulodynia and decreased arousal in women, but these findings need to be further substantiated by prospective studies [162].

Several drug classes used to treat HTN may cause ED [163,164,165]. A 2011 systematic review found that thiazides and most beta-blockers adversely affected erectile function, with the exception of the beta-blocker nebivolol, which was shown to positively influence sexual function in men. ACE inhibitors, ARBs, and CCBs had neutral or positive effects [166]. Aldosterone antagonists such as spironolactone have been demonstrated to have a negative effect on sexual function [165].

As previously described, CKD patients have a high prevalence of depression, anxiety, and neuropathic pain [167]. Iwagami et al. also found that antidepressant use in dialysis patients is one-and-a-half times higher than in the general population. Despite this, there are very few studies supporting the efficacy and the safety of antidepressant use in patients with CKD, and the evidence that does exist is generally gleaned from small non-randomized samples [168]. European Renal Best Practice 2014 guidelines recommends the first-line use of selective serotonin reuptake inhibitors (SSRIs) for up to 12 weeks in dialysis patients with moderate/major depression [169]. It is not without irony that SSRIs and selective norepinephrine reuptake inhibitors (SNRIs) are known to decrease sexual desire, cause ED in men, decrease vaginal lubrication in women, and increase difficulties with orgasm [170,171]. Of the SSRIs, paroxetine is responsible for a higher rate of SD [172]; however, all SSRIs have been associated with delayed or inhibited orgasm/ejaculation as well as reduction in libido and arousal [140]. Because of its anti-IL-6 effects, sertraline underwent clinical trials (“Study of Sertraline in Dialysis”) for the therapy of depressed HD recipients [173], and because of high drop-out rates, no significant benefit has been proven [174]. Sertraline appears to be responsible for lower rates of treatment-emergent SD (TESD) comparable to fluoxetine [175], and further investigation of its safety in depressed CKD patients is warranted.

Because of the negative effects of serotonin on sexual function, non-SSRI antidepressants may offer the solution. Rates of TESD are reportedly lower in patients who were prescribed SNRIs compared with SSRIs. To name only a few, desvenlafaxine has been shown to be promising in prospective studies; a study group of 72 depressed patients exhibited a TESD rate of 44.4%. In a study with similar methodology of 1022 patients prescribed SSRIs and other SNRIs, TESD was 66% and 75.4%, respectively [175]. Reboxetine is an antidepressant with a primarily noradrenergic mechanism of action, and it has been shown to be superior to fluoxetine in preserving the ability to achieve orgasm [176]. Bupropion, which inhibits the reuptake of norepinephrine and dopamine, is a well-known treatment alternative for depressed patients experiencing SD. Unfortunately, while their capacity for reducing depressive symptoms and preserving sexual function is promising, these agents need to be used with caution in patients with CKD. It has been shown that the elimination half-life of these and other non-SSRI antidepressants is prolonged in HD patients, and the European Renal Best Practice guidelines recommend dose-reduction, which may have implications for their antidepressant effectiveness [169]. In a phase 4 double-blind randomized, controlled trial, the novel antidepressant vortioxetine was shown to be associated with less TESD than paroxetine [177]. It appears that its pharmacokinetics are not altered by renal or hepatic impairment [178], and dose-adjustment is not necessary in the setting of CKD and ESRD [179].

It is important to assess CKD patients for depression and depressive symptoms. The use of the BDI instrument, described earlier, is valuable for the screening and the detection of depression [159,180,181]. Physicians should play a proactive role in managing this debilitating comorbid condition, which has a significant impact on sexual function and QOL as a whole. While it is not always possible to manage depression and sexual function to the highest level of satisfaction, it is important for clinicians to be aware of the drugs that are being prescribed in the course of CKD-mediated depression, especially for sexually active patients.

#### 1.6.2. Treatment of SD in Women

After completing a medication review, pharmaceutical treatment may be considered. In the re-establishment of normal female sexual function, we aim to spearhead three separate processes: resumption of menses, regulation of menses, and improving libido. Oligomenorrheic or amenorrheic women require regular gynecologic evaluation, and progesterone administered at the end of the monthly cycle can help with the resumption and the regulation of menses. Progesterone may also be important for opposing estrogenic effects on endometrial tissue [11]. In premenopausal dialyzing women who resume menses successfully, birth control is recommended. The patient should also be evaluated to ensure her menstruation does not contribute to or exacerbate anemia [11].

Pharmacological therapy for improving female libido is sorely lacking. Most treatments are unlicensed and prescribed off-label for sexual symptom clusters, and data on hormonal pharmacological therapy in premenopausal women is scant [182]. Hormone replacement therapy (HRT), that is, estrogen alone or in combination with progestogens, has been shown to be minutely to moderately effective in improving sexual function (e.g., decrease in pain) in perimenopausal women. It is suspected that only 11.3% of postmenopausal women on HD are receiving HRT, even though the benefits for bone and cardiovascular health are clear [12]. The synthetic steroid tibolone, indicated for postmenopausal symptoms, seems to have a positive effect on sexual symptoms. Classified as a selective tissue estrogenic activity regulator, it exhibits different properties in different tissues and has been shown to improve mood and enhance sexual desire through improvement of genital circulation and vaginal pulse amplitude [183,184]. Vulvovaginal atrophy (VVA) may be treated with local estrogen therapy in the form of low-dose intravaginal preparations, and this may be particularly pertinent in women receiving HD [185]. Transdermal testosterone creams/gels and systemic and intravaginal DHEA may improve dyspareunia [186]. Androgens including the transdermal testosterone patch (TTP) have also been shown to be useful in women with severe menopausal symptoms including sexual complaints. Despite evidence from randomized controlled trials showing their efficacy in improving arousal, desire, orgasm, and overall satisfaction, TTPs are not available in many countries, including the US and those of the EU. Currently, they are limited for use in surgically-menopausal women [187]. TTPs have also been investigated for use in TESD and loss of libido after SSRIs/SNRIs with promising results [188]. The selective estrogen receptor modulator (SERM) ospemifene, intended for dyspareunia in postmenopausal women with VVA, is well tolerated and appears to result in significant improvement of every sexual function domain after 12 weeks of use [189].

Novel therapies in the form of non-hormonal compounds include flibanserin, a serotonin (5-HT)1A agonist and 5-HT2A antagonist with moderate affinity for 5-HT2B, 5-HT2C, and dopamine D4 receptors. Its mixed mechanism of action facilitates the normalization of central nervous system neurotransmitter levels to enhance sexual desire [190]. Other compounds under investigation for the treatment of female SD include melanocortins, vasoactive drugs including phosphodiesterase-5 inhibitors, and oxytocin [182]. The safety profile of these biological compounds for therapeutic use in CKD patients and throughout RRT remains to be seen.

#### 1.6.3. Treatment of SD in Men

One class of drugs that may benefit male patients is phosphodiesterase-5 (PDE-5) inhibitors such as vardenafil and sildenafil. In a double-blinded trial validated by the IIEF-5 instrument, the authors found that both vardenafil and sildenafil were efficacious and well tolerated in patients receiving RRT [191]. PDE-5 inhibitors do not significantly alter the pharmacokinetics of post-transplant immunosuppressive agents nor do they interfere with allograft function [128], thus positive results have been reported with vardenafil [192] and sildenafil [193] in the treatment of ED after RT. However, tacrolimus prolongs the half-life of PDE-5 inhibitors, and appropriate dosing instructions must be emphasized to prevent hypotension [128]. Different PDE-5 inhibitors have different properties, and selection is based on cost, tolerability, and pharmacokinetic properties such as speed of onset. All PDE-5 inhibitors require dose-adjustment in patients with renal impairment [194]. Additionally, PDE-5 inhibitors may be therapeutic for hypertensive patients and may also reduce proteinuria [195].

Hormonal imbalances in advanced CKD, particularly with regard to LH and testosterone levels, lead to high rates of hypergonadotropic hypogonadism in male patients [102]; thus, the normalization of testosterone may correct the male hormonal profile, and sexual function may also see improvement. Zinc deficiency is known to be a reversible cause of gonadal dysfunction in uremic patients [196], and one strategy for improving sexual function is to supplement zinc orally or in the dialysate. Oral zinc administration alone resulted in an increase in plasma testosterone [197]; however, no appreciable change in LH and FSH levels was observed [198]. Potency, libido, and frequency of intercourse improved in patients given zinc supplementation [197], but the potential for zinc to reliably improve SD remains an open question [12].

Testosterone replacement therapy (TRT) has been shown to have positive anabolic effects on malnourished CKD patients. The hormone is administered in patch, gel, or injectable forms, and oral therapy is not recommended [12]. However, Snyder et al. [199] demonstrated in a large cohort that TRT only resulted in moderate improvement in sexual function; full restoration of sexual function was only observed in 11% of dialyzing men in a 1998 study [200]. TRT also does not normalize libido and potency in the majority of hypogonadal men, despite normalization of FSH and LH [103]. Fugl-Meyer et al. [102] showed that testosterone level is only modestly correlated with erectile function, and a review by Rastrelli et al. [201] found that TRT can be effective for milder forms of ED only. On the other hand, androgen deficiency is also correlated with increased incidence of anemia [110], and the addition of androgens to EPO therapy seemed to increase the responsiveness of erythroid progenitors to EPO [11]. In a small study, TRT was shown to improve hemoglobin and hematocrit, leading to improved QOL in men with moderate-to-severe CKD [202]. The correction of anemia has been shown to increase libido, sexual desire, and improve erectile function [32,113], and EPO therapy has been shown to improve erection quality in HD patients [40,41]. In a retrospective study, Burnett et al. [115] found that EPO administration before radical nerve-sparing prostatectomy may confer penile protective effects and improve erectile function recovery. This followed an earlier urological study that demonstrated EPO receptors in penile tissue and periprostatic neurovascular bundles and proved that EPO is a neurotrophic factor in the context of neurogenic ED [203]. The ongoing ERECT trial, placebo-controlled and double-blind, is currently investigating subcutaneous administration of EPO in men undergoing radical nerve-sparing prostatectomy [204], and positive results may have implications for the treatment of neurogenic ED in the course of CKD.

As described previously, hyperprolactinemia may reduce sexual function in men, and extreme hyperprolactinemia in men is correlated with low libido, decreased testosterone, and infertility [11]. In a randomized controlled trial conducted, daily oral vitamin E therapy was shown to decrease prolactin levels [205]. There are no further studies exploring the effect of vitamin E on prolactin levels. Trials of bromocriptine have found that this dopamine agonist successfully normalizes prolactin levels and may improve sexual function in men and women, including in mild hyperprolactinemia [206] and in patients on HD [207].

Lastly, intracorporeal injection therapy using vasodilators such as prostaglandin E1 alprostadil has proven to be effective, especially in men who do not respond to oral agents. This may be combined with papaverine and/or phentolamine or PDE-5 inhibitors as salvage therapy for severe vasculogenic ED [208]. Vacuum constriction devices and surgical implantation of penile prostheses are other options for the treatment of ED [194].

## 2. Conclusions

Renal replacement therapy has led to considerable decrease in mortality in CKD and ESRD patients. Considerations for quality of life in CKD and dialyzing patients must take into account their disproportionate prevalence of SD. There is considerable heterogeneity in the recent literature when comparing SD between different RRT techniques, and there are no clear guidelines for switching modalities for patients wishing to resume menstruation, improve libido, normalize hormonal profiles, and/or conceive. The complex and multifactorial etiology of SD is a significant obstacle, and the comorbidities that plague these patients only muddy the waters. While renal transplantation improves many aspects of sexual function and fertility, it is far from the purported silver bullet. Iatrogenic causes of SD can also be a source of frustration for clinicians and patients alike. Furthermore, the sensitivity and the stigma of SD has been a long-standing barrier to clinical advances, and many gaps exist in the research for both men and women. Female sexuality has been understudied, perhaps due to subjective and inconsistent self-reporting methods that are not as coherent with the medical model that favors objective measures, and pharmaceutical interventions for female libido are woefully limited. Patient care must increasingly focus on maintaining high QOL, and the many unknowns in the identification and management of SD in CKD patients need to be addressed in large-scale cohort studies and clinical trials. Advancements in this under-quantified field are paramount and highly anticipated.

## Figures and Tables

**Table 1:** Psychometric measures of SD validated for use in chronic kidney disease (CKD).

Validated Scale	Description	Scoring	References
Arizona Sexual Experience Scale (ASEX)	5-item clinician- or self-administered questionnaire for use in patients with psychiatric illness and/or physical health complaints Assesses sexual desire, arousal, vaginal lubrication/penile erection, ability to reach orgasm, and satisfaction from orgasm.	Each item scored from 1 to 6; final total score between 5 and 30. Diagnosis of sexual problems for: total score of ≥19; score on any one question of ≥5; scores on any three individual questions of ≥4.	[155,156]
Female Sexual Function Index (FSFI)	19-item self-reported questionnaire. Assessing all aspects of female sexual function: sexual desire, sexual arousal, lubrication, orgasm, satisfaction, and pain/discomfort.	Each domain is scored from 0 (or 1) to 5, maximum score of 36 (no SD). A score of ≤26 diagnoses female SD.	[79]
International Index of Erectile Function (IIEF)	15-item self-reported questionnaire. Evaluation of overall male sexual function: erectile function, orgasmic function, sexual desire, intercourse satisfaction, and overall satisfaction.	The Erectile Function (EF) domain can solely discriminate between men with and without ED. EF domain is categorized into 5 groups: No SD (26–30); mild (22–25); mild to moderate (17–21); moderate (11–16); and severe (6–10).	[134,153]
Sexual Health Inventory for Men (SHIM), also known as IIEF-5, the brief screening version of IIEF	Screening and diagnosis of ED and severity of ED and is reliable for patient self-assessment of ED.	Scores from 5 to 25; ED is classified into five categories based on scores: no ED (22–25); mild (17–21); mild to moderate (12–16); moderate (8–11); and severe (5–7).	[152]

SD: sexual dysfunction; ED: erectile dysfunction.

## Data Availability

No new data were created or analyzed in this study. Data sharing is not applicable to this article.

## Abbreviations

5-HT	5-hydroxytryptamine (serotonin)
AMH	anti-Müllerian hormone
BDI	Beck’s Depression Inventory
CAPD	continuous ambulatory peritoneal dialysis
CES-D	Center for Epidemiologic Studies Depression Scale
CKD	chronic kidney disease
DHEA	dehydroepiandrosterone
DM	diabetes mellitus
ED	erectile dysfunction
EPO	erythropoietin
ESRD	end-stage renal disease
FSH	follicle-stimulating hormone
FSFI	Female Sexual Function Index
GFR	glomerular filtration rate
GnRH	gonadotropin-releasing hormone
HADS	Hospital Anxiety and Depression Scale
HD	hemodialysis
HPG (axis)	hypothalamic-pituitary-gonadal (axis)
HRQOL	Health-Related Quality of Life
IDA	iron-deficiency anemia
IIEF	International Index of Erectile Function
KDQOL-SF	Kidney Disease Quality of Life short form
Kt/V	dialyzer clearance of urea over time
LH	luteinizing hormone
NHD	nocturnal hemodialysis
OH/ECW	overhydration/extracellular water ratio
PD	peritoneal dialysis
PDE5	phosphodiesterase-5
PGLI	peritoneal glucose load index
QOL	quality of life
RRT	renal replacement therapy
RRF	residual renal function
RT	renal transplantation
SF-36	Medical Outcomes Study Short Form
SD	sexual dysfunction
TESD	treatment-emergent sexual dysfunction
TRT	testosterone replacement therapy
TTP	transdermal testosterone patch
VVA	vulvovaginal atrophy
